# Endoscopic management of refractory leak and gastro-cutaneous fistula after laparoscopic sleeve gastrectomy: a randomized controlled trial

**DOI:** 10.1007/s00464-022-09748-z

**Published:** 2022-11-03

**Authors:** Said Negm, Bassam Mousa, Ahmed Shafiq, Mohamed Abozaid, Ehab Abd Allah, Adel Attia, Taha AbdelKader, Ahmed Farag

**Affiliations:** 1 Faculty of Medicine, Zagazig University, Zagazig, Egypt; 2 Shepeen alkom teaching hospital, Ministry of health, Monufia, Egypt

**Keywords:** Laparoscopic sleeve gastrectomy, Gastric fistula, Laparoscopy, Endoscopy

## Abstract

**Background:**

Gastro-cutaneous fistula is a rare complication after laparoscopic sleeve gastrectomy (LSG) with incidence of occurrence 1–2%. Most of gastro-cutaneous fistulae do not respond to conservative management and need intervention either surgically or endoscopically.

**Methods:**

This prospective randomized clinical study included referred patients who had LSG performed at our department or other centers, and complicated with post-LSG leak or gastro-cutaneous fistula between December/2019 and March/2021. Included patients were ASA Physical status I–II. Primary and secondary outcomes were recurrence of the fistula and mortality in each group after the intervention during the 18 months follow-up period, respectively.

**Results:**

Thirty patients were randomized into two groups: Surgery Group (SG, *n* = 15) and Endoscopy Group (EG, *n* = 15). Mean age of patients was 42.3 ± 8.7 and 42.6 ± 8.3 years-old in SG and EG, respectively. Females constituted 73.3% and 80% in SG and EG, respectively. Median time-to-gastric leak post LSG was six (range: 4–7) days in both groups. SG patients were surgically managed with primary repair of the gastric fistula and gastrojejunostomy in 13 patients or converting SG into Roux-en-Y gastric bypass in two patients, while EG patients were endoscopically managed with stitching, stenting, stenting and dilation, and clipping and dilation in 5, 4, 4 and 2 patients, respectively. Incidence of recurrent leak during 1^st^ week was significantly higher in SG than EG (*p* < 0.001). No mortality reported in EG, while 2 patients died in SG (*p* = 0.48).

**Conclusion:**

Endoscopic intervention may offer a successful modality in managing post-LSG gastric leak and gastro-cutaneous fistula that do not respond to conservative measures in stable patients.

Laparoscopic sleeve gastrectomy (LSG) is one of the most performed surgical procedures for treatment of morbid obesity [1, 2]. Gastric leak is the highly feared complication following LSG and its observed incidence is 1–2% [3, 4], however other post-LSG complications, such as bleeding and stricture, are less frequently encountered with a median incidence of 1.2% (range: 0.6–1.6%) [5]. Compared to post-LSG gastric leak, the incidence of gastrointestinal (GI) leak following GI oncological surgeries is 8–26% and 3–12% in distal esophagectomy and total gastrectomy, respectively [6, 7], while the incidence of gastric leak after Roux-en-Y gastric bypass (RYGB) is 2–8% [8, 9]. Post-LSG gastric leak occurs through the anastomotic suture line of the sleeve greater curvature. Leaked luminal (gastric) contents may collect next to the anastomosis (leak) or exit through the skin or the drain (fistula) [10]. Post-LSG gastric leak may occur due to impaired healing at the sleeve greater curvature suture line resulting from increased intraluminal pressure associated with gastric sleeve twist, kink or stenosis, discrepancy between tissue thickness and staple height, impaired vascular supply and uncareful use of energy sources [11]. Gastric fistula and septic shock may follow post-LSG gastric leak [12]. According to its time-to-occurrence post-LSG, gastric leaks are classified as early (on or before 3^rd^ postoperative day (POD)), intermediate (4th–7th PODs) and late (after 7th POD) [13]. Gastric leaks commonly occur between 5th and 6th PODs [13, 14]. The most common site (86%) of post-LSG leaks is the proximal gastric sleeve particularly close to the gastroesophageal junction (angle of His), however leaks in the distal gastric sleeve occur in about 14% [15]. The management of post-LSG leaks and gastro-cutaneous fistulae has not been well standardized yet [13]. It is possible to stabilize the patient and control the fistula, however, the control of leak is the most concerned issue that may pose difficulty, particularly if the leak is next to the esophagogastric junction. Patients with gastric leak who are hemodynamically unstable or in sepsis my require surgical intervention because the cost of conservative measures may be the patient’s life. Similarly, in many instances, post-LSG fistulae may not respond to conservative management, and intervention either surgically or endoscopic is usually mandated [16, 17]. Adoption of endoscopic techniques in the management of gastric leaks and gastro-cutaneous fistula has been tried in many studies [18]. Endoscopic management offers many advantages such as being less invasive, reducing septic shock and contamination, saving the time to take the proper decision, and resulting in better patient’s recovery [19, 20]. Endoscopic placement of a covered stent resulted in complete closure of post-LSG gastro-cutaneous fistula in 69–100% in early published series [21–24]. Repeat endoscopies for stent migration, retrosternal discomfort and reflux, and longer duration of external drainage were the common adverse effects associated with the use of stents in those early series [25]. Double-pigtail stent is commonly used to manage the gastric leak and permits internal drainage easing the perioperative management of gastric leak [26, 27]. A combination of covered stent and double-pigtail stent is a good option to manage gastric stenosis and associated gastric leak [26]. Endo-clips, which have been used for colonic perforation, are being used in management of post-LSG gastric leak but their role in chronic fistula is still controversial [28–30]. Lastly, endo-stitches have not been well evaluated as a primary tool for closure of post-LSG gastric leak or fistula, however it has numerous applications such as in sleeve gastroplasty, stent anchorage and closure of mucosal defects after endoscopic resections [31–35]. In this study, we compared the effectiveness of surgical versus different types of endoscopic intervention in management of post-LSG gastric leak and fistula.

## Patients and methods

### Patients

We included all bariatric patients who developed gastric leak or fistula after LSG either performed at the Department of Surgery, Zagazig University Faculty of Medicine or referred to our department between December 2019 and March 2021. While all included patients were of the American Society of Anesthesiologists (ASA) Physical status I–II, patients with ASA status III–IV or those who demonstrated a satisfactory response to the conservative measures were excluded (Patients with physical status III and IV were managed according to their general, condition, clinical status and radiological and endoscopic findings either by conservative measures or surgery). This prospective randomized controlled clinical trial was approved by Zagazig University Faculty of Medicine Institutional Review Board (Approval Number: 11130/2.12.2019) and performed in accordance with the code of ethics of the World Medical Association (Declaration of Helsinki) for studies involving human subjects. This study was retrospectively submitted in clinicaltrials.gov in May 2021 (NCT04879667). Written informed consent was obtained from all participants after explaining to them all the study procedures with its benefits and hazards.

Included patients were randomized at a 1:1 ratio to “Surgery Group, SG” or “Endoscopic Group” via the drawing of sealed envelopes containing computer-generated random numbers prepared by a third party before the start of the intervention. Sample size was calculated using open Epi program using the following data: confidence interval 95%, power of test 80%, ratio of unexposed/exposed 1, percent of patients with successful management of persistent gastric leak or fistula by surgical intervention 50% and those with successful management by endoscopy 99%, odds ratio 99%, and risk ratio 2.

Primary and secondary outcomes were recurrence of the fistula and mortality in each group after the intervention during the 3 months follow-up period, respectively.

### Diagnosis

After full history taking and complete physical examination, post-LSG gastric leak was clinically suspected and then confirmed by laboratory investigations (complete blood picture, liver and kidney functions, coagulation profile), radiological imaging (chest X-ray, computed tomography (CT) with oral and I.V contrast) and upper GI endoscopy to assess the site, size and cause of the leak. We adopted a protocol of initial radiological or laparoscopic drainage according to the amount of intraperitoneal free fluid detected by CT scan, then endoscopically inserting a stent. If the leak did not satisfactorily respond to the initial measures within 6 weeks (recommended period of 5–8 weeks by the stent’s manufacturer and asa routine in our hospital for complete healing and easy extraction of the stent), a persistent gastric leak or fistula was considered, and the patient was evaluated for eligibility to be included in this study. The included eligible patients underwent another upper GI endoscopy to reconfirm site and size of the fistula, and CT abdomen with oral and I.V contrast to determine whether the fistula had a track (gastro-cutaneous fistula) or not (gastric leak).

### Intervention

Patients, randomized to the endoscopy group, underwent endoscopic stenting (fully covered self-expanded metallic stent, FCSEMS) in case of gastric leak, endoscopic Over-The-Scope Clipping (OTSC, Ovesco Endoscopy AG, Tubingen, Germany) in case of gastric-cutaneous fistula, endoscopic suturing (OverStitch [36], Apollo Endo-surgery, TX, United States) in case of large leak or fistula size regardless of the presence of track or not, and lastly, if there is distal sleeve pouch narrowing, we combined endoscopic OTSC or OverStitch with endoscopic balloon dilation (we had six cases were diagnosed with nonfunctional strictures due to fibrosis and successfully managed with balloon dilation there were no cases managed with strictureplasty). Patients, randomized to the SG, underwent either primary repair of the fistula and gastrojejunostomy or converting LSG into Roux-en-Y gastric bypass. Primary repair and gastrojejunostomy was utilized in cases of fistula in upper 1/3 of the pouch, fistula of small size, large size sleeve pouch, old patients or patients without comorbidities. After primary closure of the fistula, a standard technique of gastrojejunostomy performed in antecolic orientation. The site of anastomosis was proximal to the site of the repaired fistula. The afferent loop is about 50 cm from duodenojejunal junction. while patients with fistula in middle and lower part of the pouch, fistula of large size, small size sleeve pouch, patients with good general conditions or patients with comorbidities were subjected to Roux en Y gastric bypass.

### Follow up after endoscopy and discharge from the hospital

All patients were clinically examined, and laboratory checked during the hospital stay. Any suspected gastric leak post repair mandated CT scan with oral and I.V contrast and upper GI endoscopy. After discharge, patients who had undergone balloon dilation as a part of their repair procedure, were endoscoped every 4 weeks to continue the dilation till relief of distal pouch narrowing. Patients were followed-up for 18 months post repair.

## Statistical analysis

Analysis of data was performed using SPSS (Statistical Package of Social Services) version 22. Quantitative variables were described as mean (± SD, standard deviation) and median (range) according to Shapiro test of normality. Qualitative variables were described as number and percentage. Chi-square test was used to compare qualitative variables between the two groups. Fisher exact test was used when one expected cell or more are less than five. Unpaired t-test was used to compare quantitative variables, in parametric data (SD < 30% of the mean). Mann Whitney test was used instead of unpaired *t*-test in non-parametric data (SD > 30% of the mean). The results were considered statistically significant when the significant probability was less than 0.05 (*P* < 0.05). *P*-value < 0.001 was considered highly statistically significant (HS), and *P*-value ≥ 0.05 was considered statistically insignificant (NS).

## Results

**Fig. 1:**
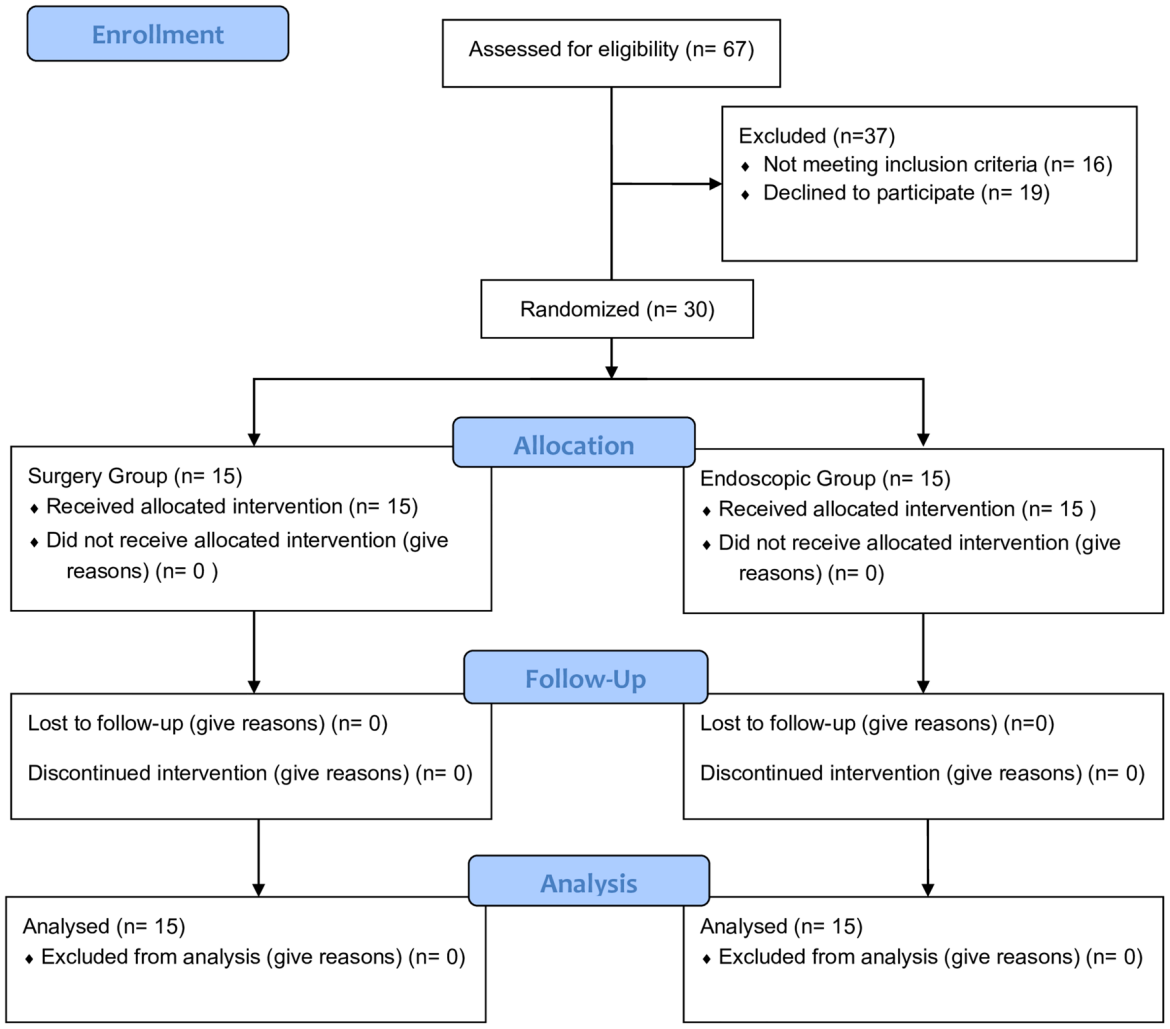
Consort flow chart

Of 67 (12 and 55 post-LSG leak or fistula patients with primary surgery performed in our department and other centers, respectively) patients who presented with post-LSG leak or fistula, 30 patients (12/30 and 18/30 with primary LSG performed in our department and other centers, respectively) met the inclusion criteria for this study. The eligible 30 patients were randomized into two groups: SG and EG (Fig. 1). the other 37 patients were excluded due to: (1) 19 patients refused to participate to study after explaining to them the protocol of management and those were managed by surgery performed by a different team (resuscitation first in ICU then surgery either classic Roux en-Ygastric bypass, primary repair and gastrojejenostomy or drainage of any collection by interventional radiology then surgery later on according to the status of each patient).; (2) 18 patients did not met the inclusion criteria as there were ASA III and IV (they were ASA I and II then became ASA III and IV just before stent insertion at the start of our protocol of leak management), some of them also presented with septic shock and unstable general condition and were also managed like the 19 patients who refused to participate.

Mean age of patients with post-LSG gastric leak or fistula was 42.3 ± 8.7 and 42.6 ± 8.3 years-old in SG and EG, respectively. Females constituted 73.3% (11/15) and 80% (12/15) of patients in SG and EG, respectively (Table 1). Patients with diabetes mellitus were 13.3% (2/15) and 20% (3/15) of SG and EG, respectively (Table 1). Gastric fistula with epithelized track was recorded in 33.3% (5/15) and 53.3% (8/15) of SG and EG, respectively (Table 1) (this is a reference to a leak controlled by a drain). Median time-to-gastric leak post LSG was 6 (range: 4–7) days in both groups (Table 1). The fundus was the most common site of gastric fistula in 80% (12/15) and 66.7% (10/15) of SG and EG patients, respectively. However, the rest of patients in both groups experienced gastric fistula at the middle of pouch greater curvature (Table 1). There was no statistically significant difference regarding gastric leak or fistula diameter between the 2 groups (*P* = 0.18); being of small (< 1 cm) diameter in 5 (33.3%) patients in each group; of moderate (1–2 cm) diameter in 60% (9/15) and 33.3% (5/15) of patients in SG and EG, respectively; of large (> 2 cm) diameter in 6.7% (1/15) and 33.3% (5/15) of patients in SG and EG, respectively (Table 1). SG patients were surgically managed with primary repair of the gastric fistula and gastrojejunostomy (86.7%, 13/15), or converting SG into Roux-en-Y gastric bypass (13.3%, 2/15), while EG patients were endoscopically managed with OverStitch (33.3%, 5/15), FCSEMS (26.7%, 4/15), dilation and FCSEMS (26.7%, 4/15), and dilation and OTSC (13.3%, 2/15) (Table 2).

**Table 1:** Characteristics of the patients

	Surgery group (*n* = 15) Mean ± SD; *n* (%)	Endoscopy group (*n* = 15) Mean ± SD; *n* (%)	*P*-value
Age (years)	42.3 ± 8.7	42.6 ± 8.3	0.42
Gender			
Male	4 (26.7)	3 (20)	
Female	11 (73.3)	12 (80)	
Body mass index	46.9 ± 3.7	45.7 ± 3.8	0.76
Comorbidities			0.9
Hypertension	2 (13.3)	2 (13.3)	
Diabetes mellitus	2 (13.3)	3 (20)	
Sleep apnea	2 (13.3)	3 (20)	
Osteoarthritis	2 (13.3)	2 (13.3)	
Infertility	0	1 (6.7)	
No comorbidities	7 (46.7)	4 (26.7)	
Time of leak post sleeve, median (range) days	6 (4–7)	6 (4–7)	0.78
Site of fistula			0.7
Fundus	12 (80)	10 (66.7)	
Middle of pouch sleeve	3 (20)	5 (33.3)	
Size of fistula			0.2
Small (< 1 cm)	5 (33.3)	5 (33.3)	
Moderate (1–2 cm)	9 (60)	5 (33.3)	
Large (> 2 cm)	1 (6.7)	5 (33.3)	
Post-LSG			0.46
Leak	10 (66.7)	7 (46.7)	
Fistula (gastro-cutaneous)	5 (33.3)	8 (53.3)

**Table 2:** Operative intervention and postoperative recurrence

	Surgery group (*n* = 15) *n* (%)	Endoscopy group (*n* = 15) *n* (%)	*P*-value
Type of intervention			
Surgical primary repair and gastrojejunostomy	13 (86.7)	0	** < 0.001**
Surgical RYGB	2 (13.3)	0	
Endoscopic stenting and dilation	0	4 (26.7)	
Endoscopic stenting alone	0	4 (26.7)	
Endoscopic clipping alone	0	0	
Endoscopic suturing	0	5 (33.3)	
Endoscopic clipping and dilation	0	2 (13.3)	
Recurrent fistula within first week			
No	1 (6.7)	15 (100)	** < 0.001**
Yes	14 (93.3)	0	

Highly significant *P*-value < 0.001 are given in bold

The observed incidence of recurrent gastric leak during the first week post-repair was significantly higher in SG than EG (*P* < 0.001); being 93.3% (14/15) and 0% (0/15) in SG and EG, respectively (Table 2). In SG, recurrent gastro-cutaneous fistula with track and gastric fistula without track (leak) occurred in 9 (60%) and 5 (33.3%) patients, respectively (Table 2). SG patients who experienced recurrence (14/15) post repair needed endoscopic management while none in EG needed further endoscopic management of the fistula post-repair. During the 18 months follow-up period, EG demonstrated no cases of recurrent gastric fistula post-repair (Table 2).

No patients died in EG, while two patients died in SG and this difference was not statistically significant (*P* = 0.48) (Table 2).

## Discussion

In general, the most frightful complication after bariatric surgery is the anastomotic leak with an incidence of 0.8–6% [37–39]. During 30-day follow-up post-LSG, the gastric leak was 0.8% [40]. In addition to identification of the site, the core principles in managing any GI fistula or leak is to drain the leaked contents and avoid further contamination by diverting the luminal contents or closure of the fistula or leak [36]. In hemodynamically stable patients, the first step to manage post-LSG gastric leak or fistula is bowel rest, percutaneous drainage, and adequate nutritional support. Failed conservative measures call for intervention whether surgically or endoscopically [41].

In our department, we perform about 500 LSG/year, and the incidence of post-LSG leaks or fistula is about 2.5%. Surgical management of gastro-cutaneous fistula after laparoscopic sleeve gastrectomy has increased incidence rates of morbidity and mortality. In this study, we experienced high failure rate of surgical intervention (93.3%) within the first week post-repair (recurrence of fistula within one week in surgical group patients was due to long remaining sleeve pouch with axial rotation or marked narrowing of the pouch so, primary repair and gastrojejunostomy are usually associated with high recurrence rates of fistula because the main cause is not corrected, with axial rotation or marked narrowing of remaining pouch, the intra-gastric pressure increases and site of fistula opens again. This can be solved by classic Roux- en- y gastric bypass but not all patients are candidates for it, and also if the fistula occurred at upper third of the remaining pouch (most common site of fistula), the recurrence rate becomes high and classic Roux- en- y gastric bypass becomes difficult in case of upper third fistula due to marked adhesions and unhealthy remaining tissue at site of fistula). Immediate surgical intervention, with abdominal washout, irrigation, wide drainage and attempts for suturing of the leak if the tissues permit, may be preferred in unstable patients with early type leak [21].

On the other hand, endoscopic intervention has become the corner stone in managing the post-LSG gastric leak or gastro-cutaneous fistula with different modalities such as stenting, clipping, balloon dilatation and endo-suturing. In our study, OTSC, along with dilation, was used in two patients (2/15 of endoscopic group), and none demonstrated clip migration or development of post-OTSC stricture over 3 months follow-up period. We started deploying the clips perpendicular to the long axis of the defect. If needed, multiple clips were placed sequentially, starting at either edge of the defect towards the center. Standard clips were passed through-the-scope to achieve superficial tissue apposition engaging the mucosa and submucosa (with 1.2 mm-wide and 6 mm-long arms capable of an approximately 12 mm grasp) and were used in conjunction with thermal ablation or mechanical scraping of the tissue around the edges of the defect to achieve a more resilient seal. In a retrospective study, OTSC demonstrated a lower success rate (50%) in managing GI fistulas in 30 patients (25/30 patients were post-bariatric surgery: 22 post-sleeve gastrectomy and three post-RYGB) with a median-time-to-OTSC delivery was 147 (range = 5–880) days [42]. Additionally, stricture post-OTSC developed after 30 days at the gastroesophageal junction in one patient who had post-sleeve gastric fistula in the previously mentioned study [42]. The authors, in the previously mentioned study, used an endoscopic cap with its diameter bigger than the defect and utilized “suction technique” that allowed better approximation of the edges with the inclusion of omentum or fat inside the clip, however, the authors did not recommend the use of graspers as it may reduce the endoscopic flexibility and suction applied at the cap [37, 38]. OTSC demonstrated a statistically significant successful closure rate for GI perforations and leaks (average 82%) compared to that of fistulas (42.9%), and long-term success of OTSC as a primary than a rescue therapeutic option (69% vs. 46.9%, respectively, *P* = 0.004) for managing GI perforations and leaks, as well [39]. A systematic review concluded that OTS clips achieved successful closure rate of 51.5% in GI fistulae and 66% in GI anastomotic leaks [40].

In this study, we used a fully covered stent (Mega stent, Taewoong Medical Industries, Gyeonggi-do, South Korea) ultra large and long (length: 24 cm, diameter: 36 mm) stent. We did not experience any complication with Mega stent, particularly migration, thanks to the design of Mega stent that fits well for the post-sleeve anatomy with reduction of migration. It completely covers the whole sleeve pouch and its lower end rests in the duodenum [43]. The reported migration incidence of FCSEMS is twice that of partially covered stents (26% vs. 13%) [44]. A case series reported the success of using Mega stent for post-sleeve leaks [45]. Mega stent demonstrated 82% success rate in closure of primary and secondary (after surgical repair) leaks following sleeve gastrectomy and RYGB, however, Mega stent use was combined with clips in selected cases in the previous study [46].

OverStitching is theoretically an optimum method of leak closure because it is the only true full thickness leak closure and performed endoscopically despite being a complex procedure. In this study, OverStitching was used in 33.3% of patients who underwent endoscopic management. The procedure began with de-epithelialization of the edges of the leak using argon plasma coagulation before applying the OverStitching system. We did not experience post-OverStitch gastric leak. Granata et al. reported 77% success rate of endoscopic management of gastric leak using direct stitches only [47]. Moreover, the same previous study demonstrated an increased success rate (85%) of endoscopic suturing combined with FCSEMS and anchoring compared to direct stitches alone [47]. In managing GI fistulae and leaks using endoscopic suturing technique, Mukewar et al. reported a 100% immediate success rate and 40% sustained clinical success rate; noting that that gastro-gastric fistulae comprised almost half of the cases in that study [48].

This study has some limitations. The small sample size that may not give powerful statistical conclusions. Exclusion of patients with ASA status > II is another limitation. Regarding the de-epithelization of the edges of fistula either for OTSC or OverStitch, there was not a single method, and it was up to the endoscopist to use argon plasma laser or mechanical scrapping of the edges. Moreover, this study showed only 3 months follow-up period. The strength of the present study is being a randomized controlled trial and comparing different endoscopic interventions on one hand with the surgical intervention on the other hand.

## Conclusion

Endoscopic intervention can be a successful modality in managing post-LSG gastric leak and gastro-cutaneous fistula without the need to surgical intervention. No recurrence leak or fistula was noted after endoscopic clipping, stitching, or stenting. Further studies with large sample size and longer follow-up period are in demand to conclude strong and valid results.

## Data Availability

All data generated during this study are included in this published article and its supplementary information files. Further minor datasets are available from the corresponding author on reasonable request.
